# Gender Parity in Geriatrics Editorial Boards

**DOI:** 10.3390/geriatrics7050090

**Published:** 2022-09-03

**Authors:** Sana Shah, Nichole B. Shumway, Emily W. Sarvis, Joe A. Sena, Alesia Voice, Aqsa Mumtaz, Abu Baker Sheikh

**Affiliations:** 1 School of Medicine, Aga Khan University, Karachi 74000, Pakistan; 2 Department of Internal Medicine, University of New Mexico Health Sciences Center, Albuquerque, NM 87131, USA; 3 Department of Internal Medicine, Montefiore St. Luke’s Cornwall Hospital, Newburgh, NY 12550, USA

**Keywords:** geriatrics, women in geriatrics, gender disparity

## Abstract

Gender equality, with an emphasis on female education, has been designated by the United Nations as one of the 17 Sustainable Development Goals (SDGs) to be completed by 2030, since gender disparity is a major impediment to scientific and economic progress. This study was carried out in an effort to address the gender gaps that can be seen in academic and scientific publications. The purpose of this study is to describe the gender distribution of editorial board members and editors-in-chief across geriatrics journals with high impact factors. Clarivate Journal Citation Reports (JCR) 2021 were used to guide the selection of geriatric and gerontology journals utilizing Scopus All Science Journal Classification Codes. The genders of the editors-in-chief and editorial board members were determined and analyzed using publicly accessible data. A total of 47 geriatric journals with an average impact factor of 4.27 were examined. Of the 65 editors-in-chief, 21 (32%) were women, whereas 876 female editorial members were found out of a total of 2414, which constitutes 36% in total. Despite making up 60% of the geriatric medical workforce, women are still underrepresented on editorial boards and as chief editors in well-known geriatric periodicals.

## 1. Introduction

More women than ever before have pursued the professions of science, technology, engineering, and mathematics during the past 50 years (STEM) [1]. Women now make up 49% of all medical students, up from 6% in 1960 [2]. However, according to data from the UNESCO Institute for Statistics (UIS), women still comprise fewer than 30% of researchers globally [1]. This suggests that they have not achieved the same advancements in research [1,3]. Despite the fact that the proportion of female first and senior authors significantly increased from 5.9 and 3.7%, respectively, in 1970 to 29.3 and 19.3%, respectively, in 2004, the total proportion of women remains low and has even plateaued in recent years [3]. 

There are more women practicing geriatric medicine than men. Currently, women make up about 60% of American Society of Geriatrics (AGS) members, 66% of AGS committee members, and 56% of the Board of Directors. [4]. Consistent with AGS membership trends, the ratio of female presidents also increased to 70% from 2009 to 2019 [4]. These results are promising, demonstrating an important surge in female representation as both practicing geriatricians and leaders in the field.

The significance of equitable gender representation in the medical sciences is emphasized by the links between gender equality in STEM and increased scientific innovation, economic growth, lower patient mortality, and improved health outcomes [1]. The measurement of women’s scholarly engagement as indicated by “authorship” in scientific publications serves as an easily accessible and objective indication of the effective integration of women in science [5]. Scientific authorship and the participation of females on editorial boards in journals reflect both their intellectual contributions and the hierarchical structures of the research community, and it is a crucial component that sheds light on the degree of the gender gap and thus serves as a proxy to identify gender discrepancies in research.

Despite recent publications analyzing the gender distributions in other medical specialties such as family medicine, gastroenterology, and orthopedic surgery, a systematic cross-sectional study of the gender proportions among editorial board members and manuscript authors in geriatric academic journals is still lacking [1,6,7]. The purpose of this study was to determine if the increased representation of women in the American Geriatric Society extends to academic and research settings.

## 2. Materials and Methods

Using the Clarivate Journal Citation Reports (JCR) 2021, we selected all the journals in the field of Geriatrics and Gerontology by using Scopus All Science Journal Classification Codes [8,9]. A third-party rating system called JCR evaluates journals on a worldwide scale using a range of different measures and indicators. We found 52 Geriatrics and Gerontology journals in total [9]. In order to identify the gender distribution among editorial board members and editors-in-chief, we looked through the online archives of each of these publications. The variables, the nation in which the journal is headquartered, and the journal’s impact factor were also collected.

For this study, gender was divided into male and female categories. We identified individual genders using publicly accessible information from academic websites and social media. When there was uncertainty or a lack of information, we used a globally recognized database called Genderize.io (https://genderize.io/ accessed on 25 March 2022) to make gender predictions using a >95% probability criterion [10].

For gender-neutral names or names for which we could not easily determine gender, we searched online for people with the exact same first name and then classified gender based on these results. To guarantee the reliability, the data were gathered by two separate investigators, and any inconsistencies were resolved by a third investigator. We were unable to obtain information on the editorial board members of five journals that were eliminated from the investigation, and a total of 47 journal data were included in the final analysis. 

In both of our interest groups, we analyzed the total number of men and women, including editorial board members and the chief editor. Descriptive analysis, such as determining the total number of male and female editorial board members and editors-in-chief, was carried out. The additional analysis includes creating pivot tables in the excel sheet in order to highlight various relationships. Moreover, the number of editors-in-chief who are men and women, divided into Europe and North America, was determined using pivot tables. This was followed by a study of the relationship between male editors-in-chief and the percentage of male and female editorial board members. We also searched for any connections between the impact factor of the journals and the gender distribution of editors-in-chief. Supplementary Tables S2–S7 show additional details.

## 3. Results

A total of 47 geriatric journals with an average impact factor of 4.27 were reviewed. Data such as each journal’s country of origin, impact factor, gender of editorial board members, and the editor-in-chief were collected. These data are presented in Table S1.

**Figure 1:**
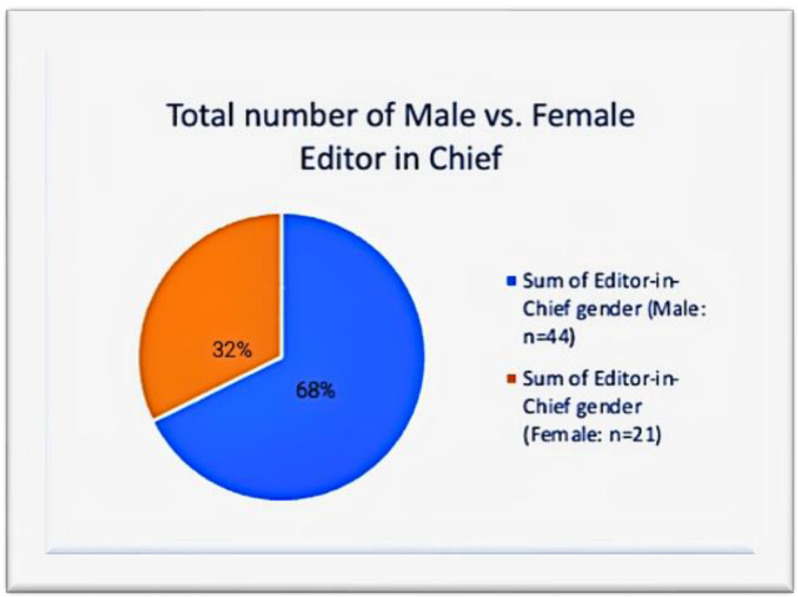
Sum of editors-in-chief.

In total, 21 of the 65 editors in chief were women. According to the pie chart in Figure 1, this accounts for around 32% of all editors-in-chief. 

**Figure 2:**
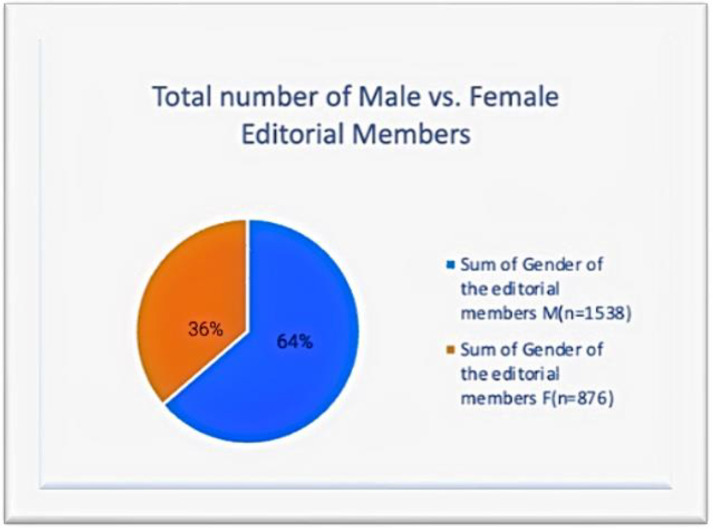
Sum of editorial members.

When the proportion of female representation as editorial members in these 47 geriatric periodicals was evaluated, there were 876 women out of a total of 2414 editorial members. The total percentage was 36%. This is included in the pie chart in Figure 2 for additional clarification.

**Figure 3:**
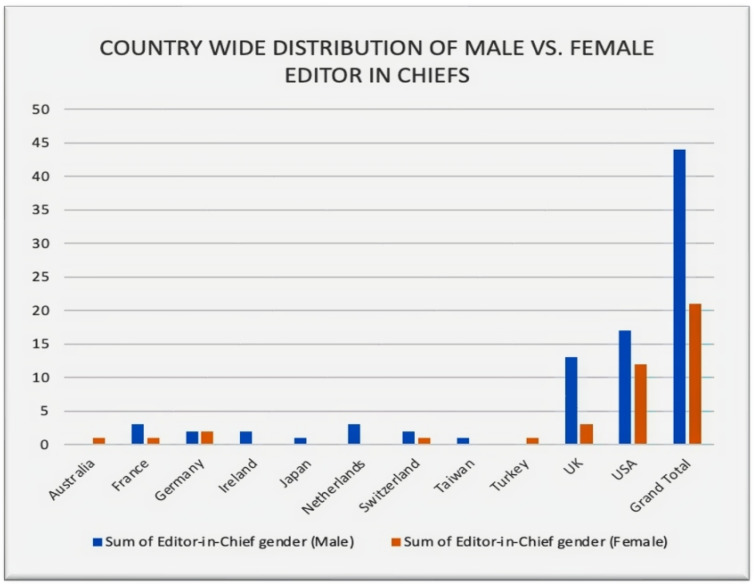
Country-wide distribution of male vs. female editors-in-chief.

The distribution of men and women as editors-in-chief among countries of publications is seen in Figure 3. The majority of the editors-in-chief (*n* = 29) were from the United States, with 41% of them being female (Table S3). Additionally, all nations had more male editors-in-chief than females, with the exception of Germany, which had an equal number of male and female editors-in-chief, and Australia and Turkey, which had a higher proportion of female editors-in-chief (100% female representation). The gender gap among editors-in-chief was most pronounced in the United Kingdom, where just 19% (3 out of 16) were female (Table S3). Furthermore, there was a total male majority in a few nations, such as Taiwan, Ireland, Japan, and The Netherlands.

**Figure 4:**
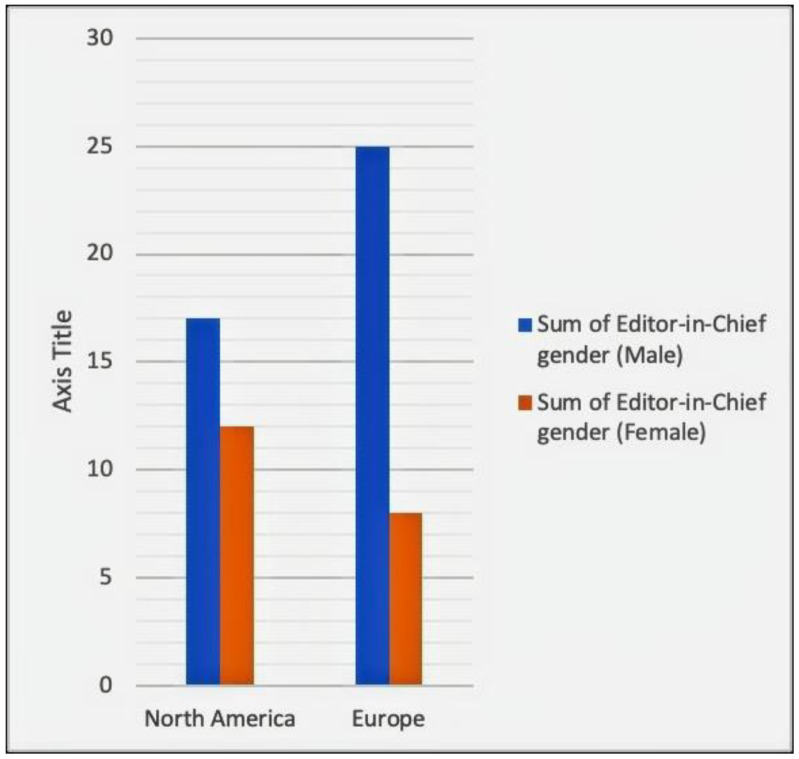
Sum of the male vs. female editors-in-chief.

Figure 4 shows the gender distribution among men and women editors-in-chief in North America versus Europe. Table S2 shows that the percentage of female editors-in-chief in North America is 41% versus 24% in Europe. This shows that there is a 1.7 times greater likelihood of a female editor-in-chief of a geriatric journal in North America as compared to Europe (41/24). The bar chart in Figure 4 further reveals this discrepancy. 

**Figure 5:**
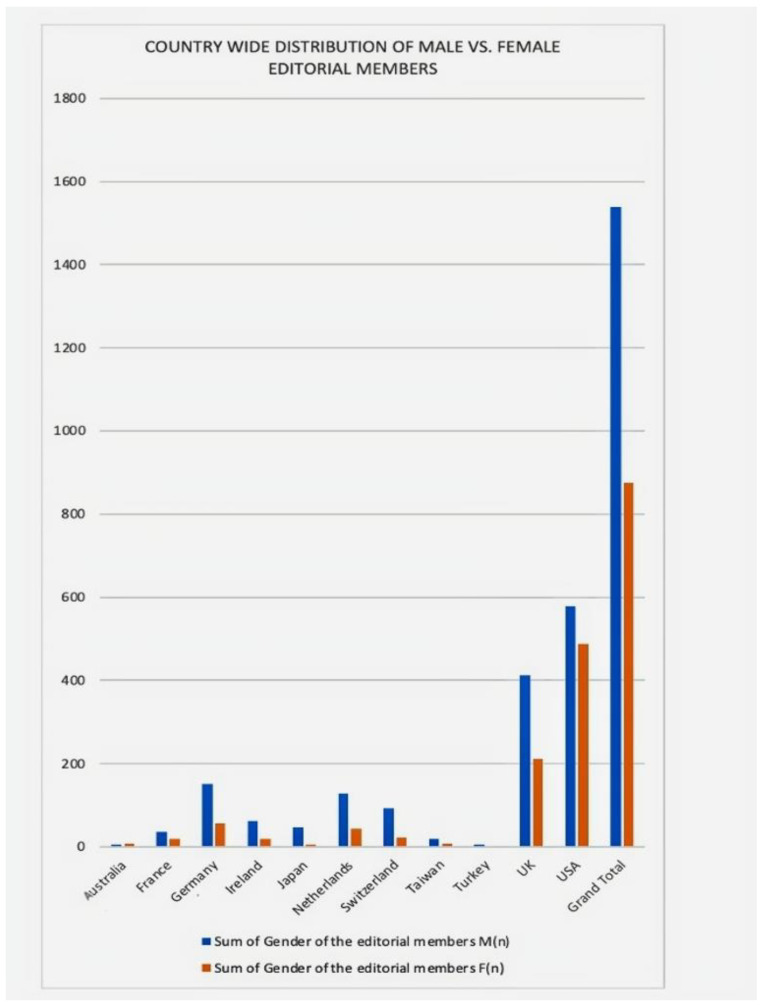
Country-wide distribution of male vs. female editorial members.

A similar country-wide distribution is shown in Table S4 for editorial members, with the majority of members (*n* = 1066) coming from the United States; 46% of these were female. Figure 5 further explains the gender discrepancy among editorial staff when it is evaluated by individual countries. With the exception of Australia, which accounts for 58% of total female editorial members, all countries have more male editorial members (7 out of 12). Furthermore, the editorial board members in Turkey are entirely male, with no female participation. This is contrasted with the female representation of editors-in-chief in Turkey, which is 100%.

Table S5 illustrates the proportion of female editors-in-chief in high-impact geriatric journals, with an impact factor of more than 10. These data demonstrate that there is an absolute male predominance when it comes to editors-in-chief of high-impact journals. 

**Figure 6:**
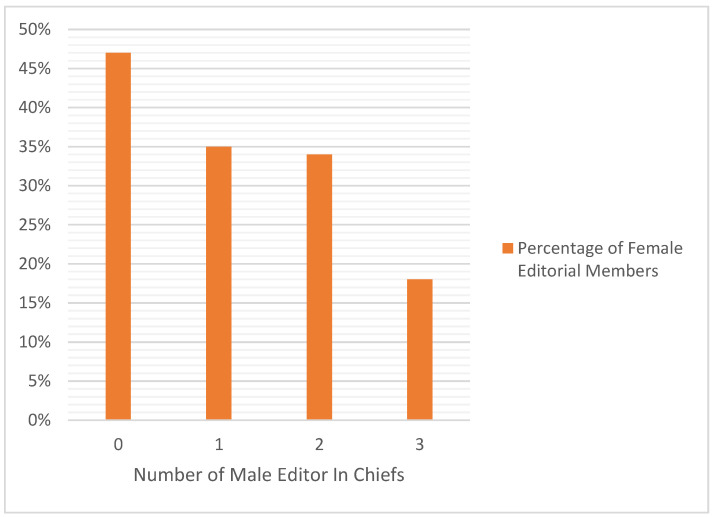
Relationship between Number of Male Editors in chief with the Percentage of Female Editorial Members.

When comparing the total number of male editors-in-chief of a journal with the percentage of female editorial member representation, there is an inverse relationship. In journals with no male editor-in-chief, females had a greater representation (47%) as editorial members as compared to journals that had three male editors-in-chief, where female editorial members dropped to 18% (Figure 6).

**Figure 7:**
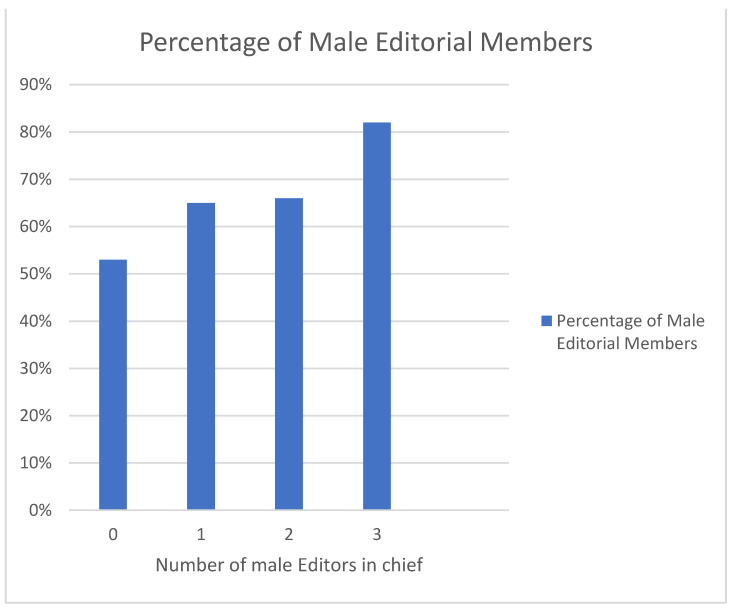
Relationship between Number of Male Editors in chief with the Percentage of Male Editorial Members.

On the other hand, the number of male editorial members has a direct relationship with the total number of male editors-in-chief as depicted in Figure 7, with the greater number of male editorial members present (82%) in journals with three male chief editors.

## 4. Discussion

This is the most thorough review of gender distribution in editorial leadership and editorial boards in academic geriatric publications to have been conducted to date, including journals from all over the world. According to our study, there are fewer women than males on editorial boards and who are editors-in-chief in the leading geriatric journals. In contrast, female physicians outnumber males in geriatrics, with women accounting for 60% of American Society of Geriatrics members [4]. Furthermore, the percentage of female presidents climbed from 70% from 2009 to 2019, in accordance with the membership as a whole [4].

These numbers present a rather perplexing situation. Although there are more women than men pursuing geriatric medicine, this does not translate to women holding leadership positions in journals or academic contributions. There is a clear gender gap. Women account for a total of 32% of editors-in-chief and 36% of editorial board members across high-impact geriatric journals. These findings for geriatric publications are not dramatically different from the results for the average female representation in research across disciplines, which indicate that women continue to account for less than 30% of researchers globally [1]. 

Another noteworthy trend is that male chief editors and female editorial board membership in journals are inversely correlated. Conversely, male editors-in-chief and male editorial board membership are positively correlated. Leung et al. highlighted this pattern in a report published in 2021 on “Gender inequalities in gastroenterology and hepatology authorship and editorial boards.” Male first and senior authorship and male-dominated editorial boards had statistically significant positive correlations, while female first and senior authorship and male-dominated editorial boards had statistically significant negative correlations [1].

The number of male editors-in chief- in journals also had a positive correlation with the number of male editorial members and an inverse relationship with the number of female editorial members. In journals with three male editors-in-chief, the percentage of male editorial members was 82%, while female editorial members made up a mere 18%. This heightened affinity between people who share traits is known as inbreeding homophily, and it appears to be a sociological population-level characteristic of human cultures [11]. The work of female scientists suffers as a result of this inbreeding homophily because of inadvertent prejudice [11]. 

Another interesting feature of this gender gap is how inadequate female participation in leadership positions in research translates to lower female representation in clinical trials and female-focused research initiatives. Throughout several centuries of medical study, the typical prototype has been male. Since the beginning of anatomical science, the bodies utilized for dissection and drawing have been primarily male [12]. This level of sex blindness in medical research and educational materials has repercussions for people and groups who do not fit the mold and may result in incorrect diagnoses, missed chances for intervention, or simply the wrong dose of the wrong medication [12]. Furthermore, there is a strong positive correlation between women’s authorship and the likelihood that a research study will include gender and sex analysis, according to a sample of more than 1.5 million medical research papers that looked at the possible link between women’s participation in medical science and attention to gender- and sex-related factors in disease-specific research [13]. These results support debates on the relationship between women’s involvement in medical research and its conclusions and demonstrate the synergistic effects of encouraging both the growth of women in science and the inclusion of gender and sex analysis in medical research [13].

A noteworthy finding of this study is the lack of female chief editors in journals with an impact factor higher than 10. This is concerning because the underrepresentation of women in high-impact journals spills over into other areas of academia, whether it is in the form of exclusive leadership roles, the distribution of substantial research funding, or a say in the formulation of health policy. This paucity of female representation in high-impact journals is also discussed by Filardo et al. [3].

With a few notable exceptions, this gender disparity was seen in the majority of the countries. The United Kingdom has the largest gender disparity among editors-in-chief, with just 19% (3 out of 16) women editors (Table S2). This corresponded to the difference between North America and Europe. In North America, the possibility of a female editor-in-chief is 1.7 times higher than in Europe as a whole. A possible explanation for this discrepancy is the overall smaller percentage of female geriatricians. For instance, of the 1222 consultant geriatricians reported in the UK in 2011, 67% were men and 33% were women [14]. This contrasts with the United States, where a staggering 60% of geriatricians are women [4]. 

It is crucial to not only pay attention to the statistics but also the whole impact that they have in order to make sure that the increase in women pursuing geriatrics and medicine correlates with gender equality in the workplace, academia, and other areas, as well as visibility, recognition, and resource allocation. This was also highlighted by Michelle Ryan in an article published in Nature in 2022 titled “To improve gender equality, utilize evidence [15].” She discusses the “glass-cliff” phenomenon, which argues that women are more likely than males to be handed difficult, insecure, and even condemned to fail leadership jobs. As a result, she challenges the standard of leadership roles granted to women as compared to men.

There are probably several underlying factors contributing to the persistently low representation of women in academia in general and as editors-in-chief in particular. Evidence suggests that women are less likely than men to accept requests to join journal editorial boards, which may be because editorial positions occasionally overlap with clinical or academic duties [16]. Furthermore, societal beliefs that still preferentially assign informal care tasks to women limit their progress. Women spend more time on unpaid childcare, housekeeping, and cooking throughout the globe [17]. In addition, career advancement may be hampered by the fact that women are more likely than males to take breaks from their jobs, such as maternity leave [17]. Unconscious gender biases may explain the undervaluing of women’s academic achievements and perpetuate the belief that women are unsuitable for key leadership positions, such as editors-in-chief [17]. These social and cultural barriers constitute a significant barrier to women’s advancement in these areas.

It is crucial to transform the workplace culture if gender parity is to be achieved in academia and in positions of power that may influence policy and change. In essence, female geriatricians need to have access to reliable mentors who will not only stand up for them but also lessen the obstacles and difficulties they encounter at work. In addition to being encouraged to have more influence in academics, women also need to receive equal compensation. As per a 2017 Washington Post analysis, women are underpaid as compared to men across a range of professions, including nursing, dentistry, and the pharmaceutical industry [4]. Policy changes that advocate for paid maternity leave and provide access to quality child care at a low cost may also motivate more women to step up and contribute to academic research and leadership in geriatrics. Furthermore, in order to obtain research funding, a clear, well-funded gender equality plan should be in place. Gender parity in geriatric medicine will translate to equitable access to health care in a country where 54.1 million, or 16% of the population, are 65 and older.

Strengths of this study include the fact that it is the most current analysis of gender in the leadership of top-ranked geriatric publications to date. The inclusion of people with names from Asia-Pacific regions is another advantage of this study, as these names are typically challenging to gender identify in gender name databases that are widely utilized.

The fundamental limitation of our study is that it is retrospective; so, no temporal inferences or trend assessments can be derived. Misclassification of gender and failure to recognize nonbinary gender are both possibilities based on our stated methodology. This study may also have some limitations because not all editors and members of a magazine must be citizens of the same nation, even if the journal’s headquarters are there. For example, a geriatrician in North America may serve as an editorial member for a European journal and vice versa. That is, a journal from one country may be entirely female or male, yet, nevertheless, include people of other nationalities.

Despite making up 60% of the geriatric medical workforce, women are still underrepresented on editorial boards and as chief editors in the major geriatric journals. Effective mentorship, workplace changes, the implementation of gender equity rules for publication funding, and assuring equal pay are all possible avenues for progress. 

## Supplementary Materials

The following are available online at https://www.mdpi.com/article/10.3390/geriatrics7050090/s1, Table S1: Supplemental Table 1, Table S2: Supplemental Table 2, Table S3: Supplemental Table 3, Table S4: Supplemental Table 4, Table S5: Supplemental Table 5, Table S6: Supplemental Table 6, Table S7: Supplemental Table 7.
