# Supplementary material for: Gender Parity in Geriatrics Editorial Boards
Source: Geriatrics (Basel). 2022 Sep 3;7(5):90. doi: 10.3390/geriatrics7050090 (PMC9498408; doi:10.3390/geriatrics7050090)
Supplement: Supplementary file 1 [file geriatrics-07-00090-s001.zip › geriatrics-1869712-supplementary.pdf]

Supplemental Table S1

| Journal | Journal Impact Factor | Country where Journal is Based | Editor-in-Chief Gender (Male) | Editor-in-Chief Gender (Female) | Gender of the Editorial Members M(n) | Gender of the Editorial Members F(n) |
|---|---|---|---|---|---|---|
| Journal of Cachexia Sarcopenia and Muscle | 12.91 | Germany | 1 | 0 | 45 | 11 |
| Aging Research Reviews | 10.895 | Ireland | 1 | 0 | 33 | 17 |
| Age and Aging | 10.668 | UK | 1 | 0 | 27 | 11 |
| Aging Cell | 9.304 | UK | 2 | 2 | 81 | 28 |
| GeroScience | 7.713 | USA | 1 | 1 | 31 | 13 |
| Journal of Gerontology Series A-Biological Sciences and Medical Sciences | 6.053 | USA | 1 | 0 | 68 | 40 |
| Frontiers in Aging Neuroscience | 5.75 | Switzerland | 1 | 0 | 5 | 2 |
| Aging-US | 5.682 | USA | 3 | 2 | 65 | 12 |
| Journal of The American Geriatric Society | 5.562 | USA | 1 | 0 | 47 | 31 |
| Mechanisms of Aging and Development | 5.432 | Netherlands | 1 | 0 | 38 | 18 |
| Aging and Disease | 5.4 | USA | 3 | 0 | 31 | 9 |
| Immunity & Ageing | 5.366 | UK | 2 | 0 | 22 | 10 |
| Biogerontology | 5.252 | Switzerland | 1 | 0 | 40 | 5 |
| Journals of Gerontology Series B—Psychological Sciences and Social Sciences | 5.06 | USA | 1 | 1 | 65 | 98 |
| BMC Geriatrics | 4.878 | UK | 1 | 0 | 103 | 82 |
| American Journal of Geriatric Psychiatry | 4.748 | USA | 1 | 0 | 21 | 13 |
| Clinical Interventions in Aging | 4.744 | UK | 1 | 0 | 15 | 11 |
| Neurobiology of Aging | 4.673 | Netherlands | 1 | 0 | 45 | 7 |
| Journal of the American Medical Directors Association | 4.669 | USA | 1 | 1 | 40 | 29 |
| Rejuvenation Research | 4.663 | USA | 0 | 1 | 39 | 11 |
| Aging and Mental Health | 4.411 | UK | 2 | 1 | 30 | 25 |
| Experimental Gerontology | 4.032 | Netherlands | 1 | 0 | 45 | 18 |
| Drugs and Aging | 3.923 | UK | 1 | 0 | 28 | 9 |
| International Psychogeriatrics | 3.878 | UK | 1 | 0 | 22 | 14 |
| Aging clinical and experimental research | 3.638 | Germany | 0 | 1 | 93 | 38 |
| Journal of Geriatric Oncology | 3.599 | USA | 0 | 1 | 35 | 45 |
| International Journal of Geriatric Psychiatry | 3.485 | UK | 1 | 0 | 44 | 7 |
| Journal of geriatric physical therapy | 3.381 | USA | 0 | 1 | 4 | 5 |

| | | | | | | |
|---|---|---|---|---|---|---|
| Archives of gerontology and geriatrics | 3.25 | Ireland | 1 | 0 | 29 | 1 |
| Journal of nutrition health & aging | 2.99 | France | 2 | 1 | 8 | 10 |
| Gerodontology | 2.98 | USA | 1 | 0 | 16 | 8 |
| Dementia and Geriatric Cognitive Disorders | 2.959 | Switzerland | 0 | 1 | 48 | 15 |
| Geriatrics & gerontology international | 2.73 | Japan | 1 | 0 | 47 | 5 |
| Journal of geriatric psychiatry and neurology | 2.68 | USA | 1 | 0 | 15 | 7 |
| Clinical Gerontologist | 2.646 | USA | 0 | 1 | 17 | 21 |
| European review of aging and physical activity | 2.517 | Germany | 1 | 1 | 14 | 7 |
| Geriatric Nursing | 2.361 | USA | 0 | 1 | 4 | 19 |
| American journal of Alzheimer's disease and other dementias | 2.305 | USA | 1 | 0 | 45 | 23 |
| International journal of older people nursing | 2.115 | USA | 0 | 1 | 3 | 24 |
| Australasian journal on ageing | 2.11 | Australia | 0 | 1 | 5 | 7 |
| Journal of Aging and Physical Activity | 1.961 | USA | 1 | 0 | 13 | 31 |
| Geriatric orthopedic surgery & rehabilitation | 1.87 | USA | 1 | 0 | 15 | 4 |
| European geriatric medicine | 1.71 | France | 1 | 0 | 27 | 9 |
| Experimental Aging Research | 1.645 | UK | 1 | 0 | 40 | 15 |
| Journal of Gerontological Nursing | 1.254 | USA | 0 | 1 | 5 | 44 |
| International Journal of Gerontology | 0.877 | Taiwan | 1 | 0 | 19 | 7 |
| Turkish Journal of Geriatrics | 0.382 | Turkey | 0 | 1 | 6 | 0 |

Supplemental Table S2

| **Row Labels** | **Sum of Editor-in-Chief Gender (Male)** | **Sum of Editor-in-Chief Gender (Female)** | **Sum of Total Editors in Chiefs** | **Percentage of Female Editor in Chiefs** |
|---|---|---|---|---|
| North America | 17 | 12 | 29 | 41.3% |
| Europe | 25 | 8 | 33 | 24.2% |

Supplemental Table S3

| **Country** | **Sum of Editor-in-Chief gender (Male)** | **Sum of Editor-in-Chief gender (Female)** |
|---|---|---|
| Australia | 0 | 1 |
| France | 3 | 1 |
| Germany | 2 | 2 |
| Ireland | 2 | 0 |
| Japan | 1 | 0 |
| Netherlands | 3 | 0 |
| Switzerland | 2 | 1 |

| | | |
|---|---|---|
| Taiwan | 1 | 0 |
| Turkey | 0 | 1 |
| UK | 13 | 3 |
| USA | 17 | 12 |
| **Grand Total** | **44** | **21** |

Supplemental Table S4

| Country | Sum of Gender of the Editorial Members M(n) | Sum of Gender of the Editorial Members F(n) |
|---|---|---|
| Australia | 5 | 7 |
| France | 35 | 19 |
| Germany | 152 | 56 |
| Ireland | 62 | 18 |
| Japan | 47 | 5 |
| Netherlands | 128 | 43 |
| Switzerland | 93 | 22 |
| Taiwan | 19 | 7 |
| Turkey | 6 | 0 |
| UK | 412 | 212 |
| USA | 579 | 487 |
| **Grand Total** | **1538** | **876** |

Supplemental Table S5

| Journal Impact Factor | Sum of Editor-in-Chief Gender (Male) | Sum of Editor-in-Chief Gender (Female) |
|---|---|---|
| 12.91 | 1 | 0 |
| 10.895 | 1 | 0 |
| 10.668 | 1 | 0 |

Supplemental Table S6

| Number of Male Editor in Chiefs | Percentage of Female Editorial Members |
|---|---|
| 0 | 47% |
| 1 | 35% |
| 2 | 34% |
| 3 | 18% |

Supplemental Table S7

| Number of Male Editor in Chiefs | Percentage of Male Editorial Members |
|---|---|
| 0 | 53% |
| 1 | 65% |
| 2 | 66% |
| 3 | 82% |
